# A Simple Machine Learning-Based Quantitative Structure–Activity Relationship Model for Predicting pIC_50_ Inhibition Values of FLT3 Tyrosine Kinase

**DOI:** 10.3390/ph18010096

**Published:** 2025-01-14

**Authors:** Jackson J. Alcázar, Ignacio Sánchez, Cristian Merino, Bruno Monasterio, Gaspar Sajuria, Diego Miranda, Felipe Díaz, Paola R. Campodónico

**Affiliations:** Centro de Química Médica, Facultad de Medicina Clínica Alemana, Universidad del Desarrollo, Santiago 7780272, Chile

**Keywords:** FLT3 inhibitors, ligand-based drug design, computer-aided drug design, QSAR modeling, AML treatment

## Abstract

**Background/Objectives:** Acute myeloid leukemia (AML) presents significant therapeutic challenges, particularly in cases driven by mutations in the FLT3 tyrosine kinase. This study aimed to develop a robust and user-friendly machine learning-based quantitative structure–activity relationship (QSAR) model to predict the inhibitory potency (pIC_50_ values) of FLT3 inhibitors, addressing the limitations of previous models in dataset size, diversity, and predictive accuracy. **Methods:** Using a dataset which was 14 times larger than those employed in prior studies (1350 compounds with 1269 molecular descriptors), we trained a random forest regressor, chosen due to its superior predictive performance and resistance to overfitting. Rigorous internal validation via leave-one-out and 10-fold cross-validation yielded $Q^{2}$ values of 0.926 and 0.922, respectively, while external validation on 270 independent compounds resulted in an R^2^ value of 0.941 with a standard deviation of 0.237. **Results:** Key molecular descriptors influencing the inhibitor potency were identified, thereby improving the interpretability of structural requirements. Additionally, a user-friendly computational tool was developed to enable rapid prediction of pIC_50_ values and facilitate ligand-based virtual screening, leading to the identification of promising FLT3 inhibitors. **Conclusions:** These results represent a significant advancement in the field of FLT3 inhibitor discovery, offering a reliable, practical, and efficient approach for early-stage drug development, potentially accelerating the creation of targeted therapies for AML.

## 1. Introduction

Acute myeloid leukemia (AML) represents a formidable challenge in oncology, characterized by the uncontrolled proliferation of clonal cells within the hematopoietic system and leading to extensive tissue infiltration and disease progression. A pivotal player in the pathophysiology of AML is the Fms-like tyrosine kinase 3 (FLT3) receptor gene [1]. This gene, when mutated, particularly through internal tandem duplications (ITDs), confers a significant proliferative advantage to leukemic cells by activating multiple signaling pathways crucial for both disease progression and prognosis. Consequently, FLT3 mutations are a key factor in the aggressive nature of AML and have been extensively studied for their role in disease development and outcomes [2,3,4].

For decades, the treatment landscape for AML has predominantly relied on a conventional regimen consisting of continuous infusion of cytarabine combined with anthracycline drugs [5]. The effectiveness of this traditional approach is influenced by factors such as the genetic profile of the leukemia and patient age, with older patients often exhibiting markedly lower response rates. This realization has underscored an urgent need for novel therapeutic strategies capable of improving outcomes across different patient demographics [3]. In response, the AML treatment paradigm has evolved with the introduction of FLT3 inhibitors such as midostaurin and gilteritinib, targeting specific mutations, along with sorafenib and quizartinib. This shift toward customized therapy, including new combinations like CPX-351 and gemtuzumab ozogamicin, reflects ongoing efforts to align treatment strategies with the molecular characteristics of the disease [6]. The advent of targeted therapies and the expansion of the treatment arsenal signify a substantial step toward more effective and personalized treatment approaches for AML, offering hope for improved outcomes across a broader range of patient groups [7,8,9].

The understanding and development of FLT3 inhibitors have greatly benefited from the implementation of quantitative structure–activity relationships (QSARs) and molecular docking [10,11,12,13,14,15,16]. One study by Sandoval et al. [16] exemplified the use of QSARs in accurately predicting the anti-leukemic activity of compounds through linear discriminant and multilinear regression analyses. Similarly, Shih and Bhujbal et al. [11,13] identified key structural features and designed novel compounds with enhanced FLT3 inhibitory activity by integrating molecular docking with 3D-QSAR approaches. Ghosh et al. [15] further demonstrated the efficacy of computational modeling, including molecular dynamics and 3D-QSAR, in elucidating the structure–activity relationships of FLT3 inhibitors. These methodologies, endorsed by studies such as those of Fernandes and Islam et al. [14,17], have provided invaluable insights into the molecular interactions and binding affinities of potential FLT3 inhibitors, emphasizing the significance of these approaches in AML drug discovery and development.

In recent years, machine learning (ML) has gained prominence in AML subtype classification, showcasing diverse applications and high diagnostic accuracy. Liu et al. [18] developed a random forest model for the automatic classification of AML-M1 and M2 subtypes from bone marrow smear images, achieving 99.8% accuracy. Abhishek et al. [19] leveraged deep learning to classify AML alongside other leukemias, reaching 97% accuracy in binary tasks and 95% in three-class tasks. Monaghan et al. [20] applied ML to flow cytometry data, achieving 94.2% accuracy in distinguishing acute leukemias from non-neoplastic cytopenias. Awada et al. [21] integrated genomic data using Bayesian latent class methods, identifying novel AML molecular subclasses with 97% cross-validation accuracy. Similarly, Dese et al. [22] utilized support vector machines for robust image segmentation and leukemia classification, achieving 97.69% accuracy while reducing the diagnostic time from 30 min to under 1 min. Talaat et al. [23] optimized convolutional neural networks (CNNs) for leukemia detection, reporting 99.99% accuracy with hyperparameter optimization. These studies underscore the transformative potential of ML in AML diagnostics, complementing traditional methodologies and paving the way for enhanced diagnostic workflows.

Building on these advancements, ML has also revolutionized drug discovery, particularly in identifying and predicting kinase inhibitors, including those targeting FLT3. Nasimian et al. [24] demonstrated the potential of a machine learning-based model in predicting drug sensitivity, revealing crucial insights into AXL dependency in AML. Janssen et al. [25] introduced the Drug Discovery Maps (DDM) model, employing algorithms like t-SNE to visualize and predict interactions across the kinase family and leading to the discovery of potent FLT3 inhibitors. Furthermore, Zhao et al. [26] applied ML methods to classify and analyze the structure–activity relationships of a vast number of FLT3 inhibitors, uncovering key structural features associated with high inhibitory activity. These advancements, as discussed by Eckardt et al. [27], highlight the growing importance of ML in managing AML, from diagnosis to therapy optimization. Such ML-based approaches offer a promising avenue for enhancing the efficacy and precision of FLT3 inhibitor development, signifying a paradigm shift in AML treatment strategies.

Despite significant strides in FLT3 inhibitor development, unresolved challenges persist, notably in the predictive accuracy of current QSAR models. These models often exhibit limited predictive performance, underscoring the need for enhanced precision and simplicity. A common limitation is their reliance on a narrow range of molecular data for model training, which hampers their ability to generalize findings across a broader chemical space. The lack of molecular diversity and heterogeneity in these datasets limits their capacity to fully capture the complexity of potential FLT3 inhibitors. Furthermore, the notable absence of user-friendly models capable of providing rapid and reliable results stresses the necessity for more practical and trustworthy methodologies in drug discovery.

In response to these challenges, our research introduces an innovative QSAR-ML model trained on a more extensive and diverse dataset, encompassing a wider range of molecules to improve robustness and generalizability. By integrating advanced machine learning techniques with sophisticated molecular descriptors, this model aims to surpass the predictive limitations of current QSAR models. Additionally, our QSAR-ML model is designed for user accessibility, offering quick and reliable outcomes. This approach promises to enhance the identification of new FLT3 inhibitors for AML treatment, setting a precedent for more efficient and accessible drug discovery tools. Ultimately, it has the potential to revolutionize the development of FLT3 inhibitors and accelerate progress toward more effective, personalized AML treatments.

## 2. Results and Discussion

### 2.1. Molecular Diversity of the Dataset

**Figure 1:**
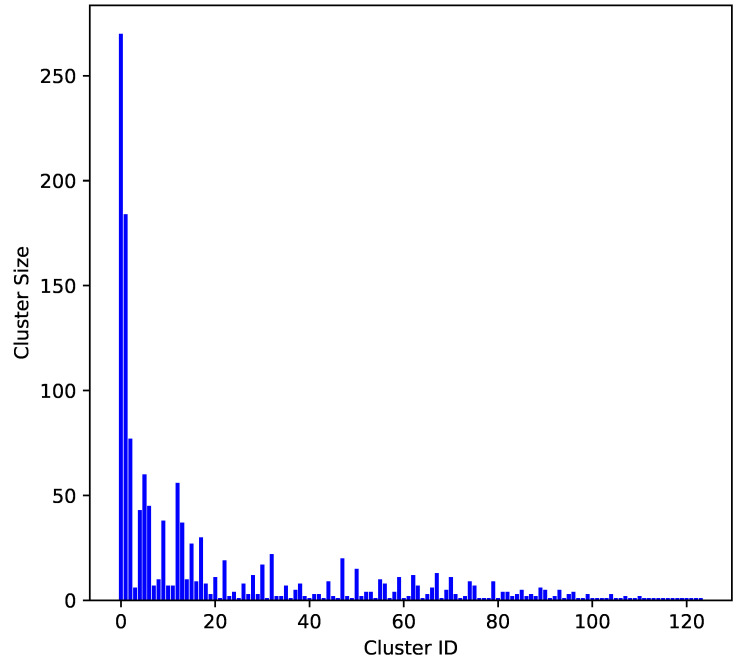
Molecular diversity clustering analysis.

To evaluate the molecular diversity of the dataset, a clustering analysis was performed using RDKit [28] to calculate the MACCS key fingerprints [29] for each molecule. The clustering algorithm applied was Butina [30], with a Tanimoto similarity threshold of 0.3, indicating that molecules within the same cluster had a similarity value of at least 0.7. The distribution of molecules across clusters is illustrated in Figure 1.

Figure 1 presents the clustering results of the molecular dataset based on the MACCS key fingerprints and the Butina clustering algorithm [29,30]. The x axis represents the cluster IDs, while the y axis shows the number of molecules in each cluster. Larger clusters indicate groups of molecules with high structural similarity, suggesting redundancy in the dataset. Smaller clusters represent more unique molecular structures, indicating greater diversity.

The analysis revealed a balanced distribution of molecular similarities and diversities, indicating that the dataset encompassed both highly similar and uniquely diverse molecular structures. The cluster with the highest similarity comprised 20% of the total molecules, while the second largest cluster accounted for 13.6%. The remaining clusters each contained less than 6% of the total molecules. This dataset contained 124 clusters, which is greater than the total number of molecules used in previous studies [10,11,12,13,14,15]. This highlights the extensive diversity employed in this work compared with prior research, offering a broader chemical space for analysis and model development. This diversity is crucial for developing robust and generalizable machine learning models for predicting the activity of FLT3 inhibitors in AML treatment.

### 2.2. Benchmarking Machine Learning Methods

This study evaluated the performance of different machine learning models built from a single dataset to predict pIC_50_ values for 1350 FLT3 tyrosine kinase inhibitors based on 1269 descriptors. The models compared included the random forest regressor (RFR), gradient boosting regressor (GBR), kernel ridge regression (KRR), Gaussian process regressor (GPR), bagging with random forest (BRF), and two artificial neural network (ANN) architectures implemented using Keras (ANN-K) and PyTorch (ANN-P).

Table 1 presents a comprehensive comparison of the machine learning models in predicting the pIC_50_ values of FLT3 tyrosine kinase inhibitor compounds across various metrics, including the R^2^ value, MAE, SD, and RMSE, for both the training and testing datasets.

### 2.3. Model Performance Overview

#### 2.3.1. Training Performance

The training performance of the machine learning models (see Table 1) was evaluated on 1080 compounds using metrics such as the R^2^ value, MAE, SD, and RMSE, providing insights into their effectiveness. The RFR and ANN-K models distinguished themselves with exceptional R^2^ scores of 0.988. Both models demonstrated remarkable precision, evidenced by low MAE, SD, and RMSE values (0.082, 0.102, and 0.102 for RFR; 0.070, 0.101, and 0.103 for ANN-K, respectively). This indicates that both models possess the ability to capture the variability in the training data across predictions.

The GBR also demonstrated high efficacy, with an R^2^ value of 0.973, positioning it as a robust model, though it exhibited a slightly higher MAE (0.126) and a broader spread for the SD (0.154) and RMSE (0.154) compared with the RFR and ANN-K (see Table 1). This indicates that while the GBR was generally accurate, its predictions were not as consistently close to the actual values as those of the RFR and ANN-K.

Conversely, KRR and the GPR exhibited moderate-to-low R^2^ values of 0.546 and 0.641, respectively (Table 1). These suggest a weaker ability to predict the training data accurately. The relatively high MAE, SD, and RMSE values for these models (0.489, 0.638, and 0.638 for KRR; 0.469, 0.526, and 0.568 for the GPR, respectively) further illustrate this point, indicating not only larger average errors but also greater variability in these errors.

Overall, while the RFR and ANN-K showed promise for applications requiring high reliability and accuracy, the GBR remains a viable choice for scenarios where a slight decrease in prediction accuracy is acceptable. In contrast, KRR and the GPR might require further tuning or reconsideration of their applicability. This analysis highlights the importance of selecting the right model based on specific performance metrics and the particular needs of the deployment environment.

#### 2.3.2. Testing Performance

When the machine learning models were evaluated using the same metrics on external and independent data (270 compounds)—which were not included in the training dataset—both the RFR and GBR exhibited strong testing performance, with R^2^ values close to 0.94 (see Table 1). This underscores their robustness in handling new data. Additionally, their low MAEs (0.197 for the RFR and 0.195 for the GBR) and RMSEs (0.246 for the RFR and 0.239 for the GBR) further highlight their precision and reliability in making predictions. These results suggest that ensemble methods, particularly those based on decision trees, are well suited for predicting pIC_50_ values for FLT3 inhibitors using the QSAR approach.

In contrast, the GPR showed significantly poorer performance during the testing phase, with a negative R^2^ value of −0.228. This indicates not only a failure to generalize but also worse performance than a model which would simply predict the mean of the dataset, likely due to overfitting to the training data or inappropriate model assumptions for the type of data used. The exceedingly high MAE and RMSE values for the GPR confirm this, reflecting large prediction errors and high variability, which diminish its practical utility.

Meanwhile, KRR exhibited moderate performance, with an R^2^ value of 0.592 (Table 1). Although better than the GPR, it still fell short compared with the RFR and GBR, suggesting that while it captured some of the variance in the data, it was not as effective or reliable. The artificial neural networks, implemented in both Keras (ANN-K) and PyTorch (ANN-P), also showed significant performance declines from training to testing, with R^2^ values of 0.907 and 0.895, respectively, coupled with higher error metrics. This discrepancy underscores overfitting as a critical challenge. Despite their theoretical strength in handling nonlinear feature spaces, ANNs are particularly susceptible to overfitting in datasets with noise or correlated features, requiring careful regularization and feature selection. In contrast, the RFR demonstrated robust performance by inherently addressing overfitting through ensemble averaging and efficiently handling correlated and noisy descriptors via its randomized feature selection mechanism.

This varied testing performance across models highlights the critical importance of selecting and tuning models based on their ability to not only fit the training data but also generalize well to new, unseen data. The RFR and GBR stood out as the more reliable models for consistent application, while the use of the GPR, KRR, and to some extent the ANN models might require more careful handling to ensure robustness and accuracy in practical applications.

### 2.4. The Model

**Figure 2:**
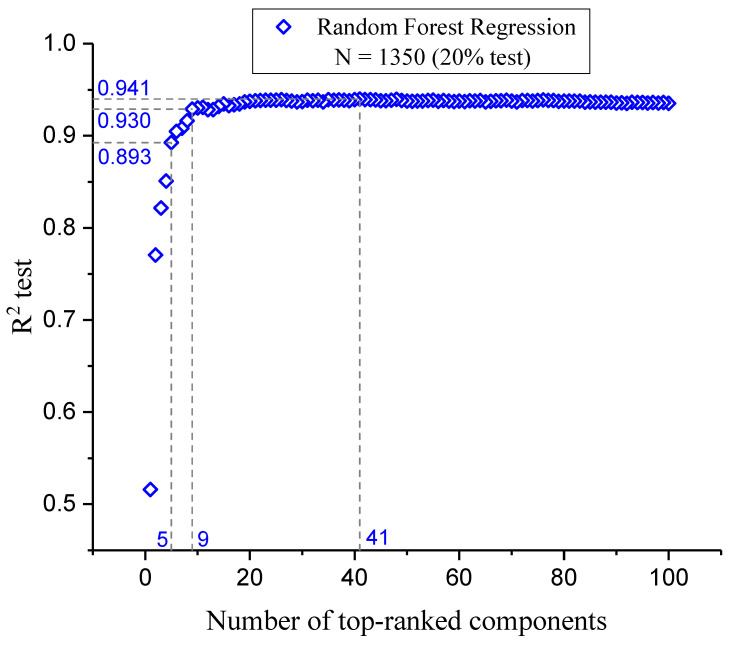
Variation in R^2^ test values as a function of the number of descriptors, ranked from most to least significant.

This section presents an analysis of the component optimization through feature selection, focusing on the efficacy of the RFR as the chosen method for predicting FLT3 tyrosine kinase inhibitor activity. The findings, illustrated in Figure 2, stress the paramount importance of the initial five molecular descriptors. These descriptors exhibited a combined R^2^ test score of 0.893, indicating their critical role in model accuracy and interpretability (see “Model Interpretation” below). The subsequent inclusion of descriptors up to the ninth one markedly improved the R^2^ test score to 0.930, with a significant but diminishing return on predictive performance with each additional descriptor. Beyond the incorporation of 41 descriptors, the R^2^ test score plateaued at 0.941, suggesting that the further addition of descriptors does not substantially enhance the model’s predictive capability.

**Figure 3:**
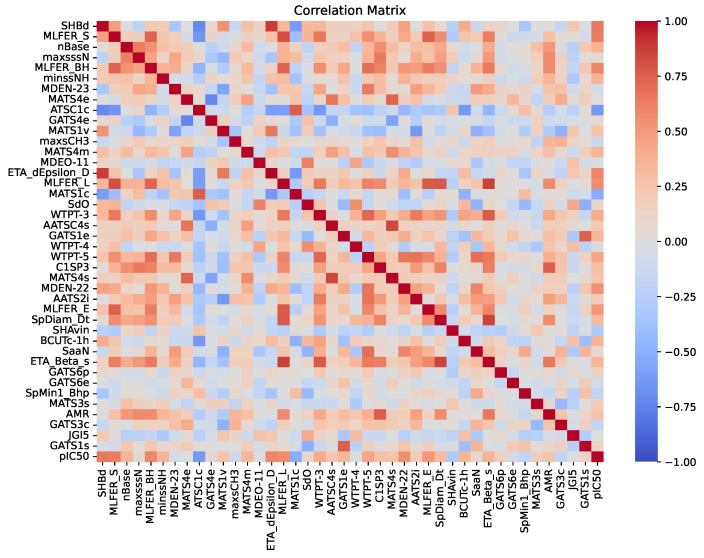
Correlation matrix of 41 descriptors and inhibitory activity (pIC_50_). The heatmap displays the pairwise correlation coefficients among the descriptors, including their relationships with the dependent variable pIC_50_. The color palette ranged from −1 (dark blue), indicating a strong negative correlation, to 1 (dark red), indicating a strong positive correlation. Neutral correlations are represented in white.

An essential aspect of this analysis is understanding the intercorrelations among the descriptors, as illustrated in the correlation matrix in Figure 3. This matrix displays the pairwise correlation coefficients among the 41 descriptors and their relationships with the inhibitory activity (pIC_50_). The color palette ranged from −1 (dark blue), indicating a strong negative correlation, to 1 (dark red), indicating a strong positive correlation, with neutral correlations represented in white. A threshold of $\left|0.90\right|$ was used to identify highly correlated descriptor pairs, which were subsequently removed during model construction. As a result, Figure 3 only contains correlations of less than $\left|0.90\right|$. Identifying and removing these highly correlated pairs is crucial, as descriptors with correlations above this threshold may contribute redundant information to the model. By examining these correlations, the selected descriptors provided unique and valuable contributions to the predictive performance of the RFR model.

This observation emphasizes the effectiveness of the RFR in capturing the complex nonlinear relationships between a manageable number of descriptors and pIC_50_ values, thus optimizing the balance between model simplicity and predictive accuracy. This outcome reaffirms the superiority of ensemble methods like the RFR in handling high-dimensional data [31] while underlining the importance of a judicious feature selection process in the development of efficient and reliable predictive models for drug discovery applications.

**Figure 4:**
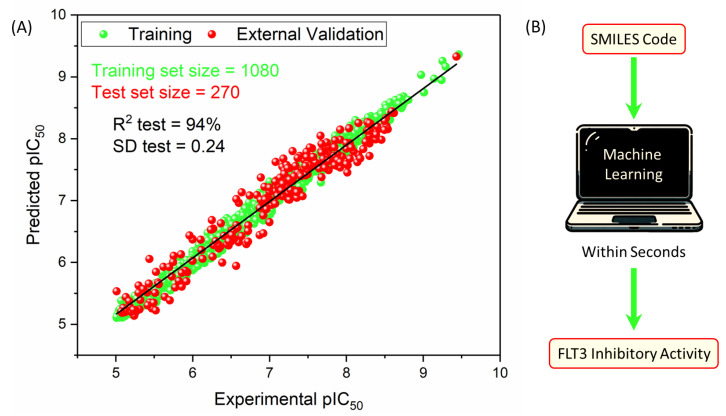
(**A**) Scatter plot illustrating the performance of the random forest regressor (RFR) model. The model was trained with 1080 compounds (green points) and externally tested on 270 compounds (red points). The plot shows the correlation between the predicted and experimental pIC_50_ values for FLT3 tyrosine kinase inhibitors. (**B**) Illustration of the developed tool for predicting pIC_50_ values of FLT3 tyrosine kinase inhibitors.

The results shown in Table 2 and illustrated in Figure 4A demonstrate the predictive capability of the RFR model for FLT3 tyrosine kinase inhibitor compounds when optimized with 41 descriptors. With a remarkable R^2^ value of 0.989 for training and 0.941 for testing, the model showcased exceptional accuracy and robustness in capturing the complex relationships between the descriptors and the pIC_50_ values of 270 inhibitors. The error metrics, including the MAE, SD, and RMSE, further affirmed the model’s precision across both the training and test datasets. The $Q_{\mathrm{LOO}}^{2}$ value of 0.926 and $Q_{10\;\mathrm{fold}}^{2}$ value of 0.922 indicate notable predictive reliability through cross-validation, emphasizing consistency in the model.

### 2.5. Comparative Analysis: QSAR Modeling

The RFR model, employing 41 descriptors, was compared to prior QSAR studies which examined FLT3 tyrosine kinase inhibitors, as detailed in Table 3. It significantly improved the accuracy of predicting pIC_50_ values for unseen compounds, achieving an R^2^ value of 0.941 and an SD of 0.237 in the test set. These results are notable considering that previous studies achieved an R^2^ value of no more than 0.891 and an SD of at least 0.3. The enhanced performance of this model not only reflects its increased accuracy but also its application to a broader and more diverse dataset of 270 compounds, which is critical for reliable predictions of FLT3 tyrosine kinase inhibition. Moreover, the $Q_{\mathrm{LOO}}^{2}$ value of 0.926, in contrast to 0.802 or lower in earlier studies, suggests that the predictive accuracy of the model is not overly dependent on specific data points or features compared with models prior to this work.

These findings underscore the effectiveness of a design which relies exclusively on the characteristics of the ligand when supported by a large and diverse dataset, rendering the ligand-based model a practical and dependable tool.

### 2.6. Model Interpretation

The interpretability of the model could be achieved by conceptually analyzing the five descriptors which were most influential on its performance. The five descriptors, detailed in Table 4, are recognized for their paramount importance: SHBdb, MLFER_S, nBase, MaxsssN, and MLFER_BH.

#### 2.6.1. SHBd

**Figure 5:**
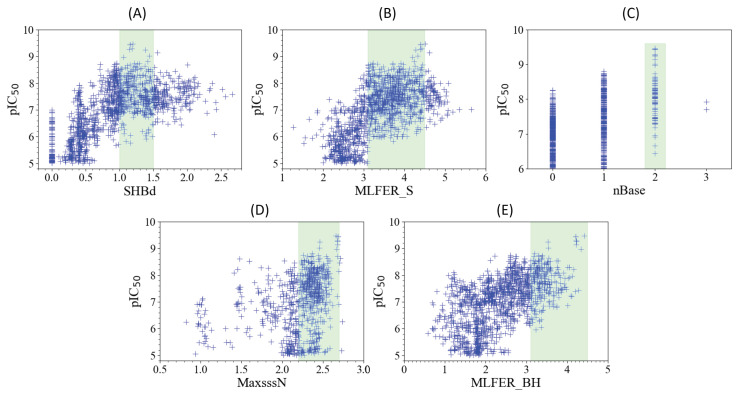
Scatter plots (**A**–**E**) showing the relationship between the five key molecular descriptors (SHBd, MLFER_S, nBase, MaxsssN, and MLFER_BH) and the FLT3 inhibitor potency (pIC_50_) of molecules in both the training and test sets. The green shaded regions in each plot indicate descriptor ranges associated with higher potency values.

The relationship between the SHBd values and pIC_50_ scores, as depicted in Figure 5A, reveals the nuanced interplay crucial for the design of FLT3 tyrosine kinase inhibitors. SHBd reflects the presence and quality of hydrogen bond donors, which are essential for stabilizing interactions within the active site of FLT3. Hydrogen bond donors facilitate key interactions with residues such as Cys694 and Cys695 in the hinge region [13]. Achieving optimal inhibitory activity requires SHBd values to be within the range of 1–1.5. Deviations from this range lead to diminished efficacy, as both insufficient and excessive hydrogen bonding capabilities can adversely affect inhibitor performance. This observation aligns with the findings of Kar et al. [10], emphasizing the need to modulate hydrogen bond interactions to avoid reduced specificity or oversaturation. By guiding the adjustment of the hydrogen bond donor capacity to a targeted range, SHBd plays a pivotal role in enhancing the potency and selectivity of inhibitors.

#### 2.6.2. MLFER_S

An analysis of Figure 5B revealed the optimal MLFER_S range for FLT3 tyrosine kinase inhibitors to be between 3.1 and 4.5. MLFER_S quantifies solvophobic energy contributions, representing hydrophobic interactions critical for binding within the hydrophobic pocket of FLT3, which includes residues such as Phe830 and Tyr693 [13]. Within this interval, the compounds exhibited peak inhibitory efficacy, while the values outside of this range resulted in decreased performance. These findings are consistent with those of Shih et al. [11], who demonstrated that moderate hydrophobic interactions enhance ligand affinity and specificity. This descriptor highlights the role of solvophobic contributions in determining the effectiveness of FLT3 inhibitors and underscores the importance of balancing solubility and hydrophobicity for optimal inhibitor design.

#### 2.6.3. nBase, MaxsssN, and MLFER_BH

The observed trends in nBase, MaxsssN, and MLFER_BH, as depicted in Figure 5C–E, respectively, collectively underscore the intricate relationship between molecular structure and FLT3 inhibitory activity.

A peak in inhibitory activity was observed when the molecules contained two basic groups (see Figure 5C). Basic groups enhance interactions with FLT3 through electrostatic attractions and hydrogen bonding with residues such as Asp698 and Lys644 [12]. This aligns with the findings of Kar et al. [10], which highlight the importance of basic nitrogen-containing groups in stabilizing ligand–receptor interactions.

MaxsssN reflects the presence of nitrogen atoms with three single bonds, such as in amine or amide groups, which are critical for forming key hydrogen bonds and electrostatic interactions. Compounds exhibiting MaxsssN values greater than 1.5 showed enhanc activity, with a more pronounced effect beyond 2.2, as demonstrated in [11]. These nitrogen configurations contribute significantly to ligand binding and specificity within the FLT3 active site.

Meanwhile, MLFER_BH extends the discussion to encompass the overall hydrogen bond acceptor capacity of the molecule. Compounds with MLFER_BH values greater than 3.1 exhibit the best inhibitory activity, as acceptors such as carbonyl oxygens and heterocyclic nitrogens form stable hydrogen bonds with critical residues like Gly697 and Cys695 [15]. This descriptor broadens the scope from nitrogenous groups to all potential hydrogen bond acceptors, suggesting that the ability to engage in hydrogen bonding is fundamental to the inhibitory mechanism.

Collectively, these descriptors—nBase, MaxsssN, and MLFER_BH—capture the essential chemical and biological factors influencing FLT3 inhibition, including electrostatic effects, hydrogen bonding, and hydrophobic interactions. These insights are supported by findings from multiple studies [10,11,12], reinforcing the relevance of these descriptors in optimizing therapeutic compounds.

### 2.7. Novel FLT3 Inhibitors Identified by Ligand-Based Screening

After applying ligand-based virtual screening (LBVS) using our customized cheminformatics model, a series of promising compounds with potential inhibitory effects against FLT3 tyrosine kinase was identified. The top five are presented in Table 5. This approach enabled the selection of new candidates exhibiting structures similar to Gilteritinib, a next-generation inhibitor [37]. This methodology highlights the utility of LBVS in efficiently identifying compounds with desired biological activity without direct physical interactions with the biological target. The identification of these pyrazinecarboxamide derivatives, with pIC_50_ values close to that of Gilteritinib (9.39) [38], emphasizes the potential of this computational approach in the discovery and development of new FLT3 inhibitors for treating AML with FLT3 mutations. These findings expand our understanding of the structure–activity relationships of FLT3 inhibitors and provide a tool for the future experimental validation of these compounds.

The relationship between the compounds identified in Table 5 and the SHBd, MLFER_S, nBase, MaxsssN, and MLFER_BH descriptors highlights the connection between substructure and potency. In the identified molecules, such as 6-Ethyl-3-[3-methoxy-4-[4-(1-methylpiperidin-4-yl)piperazin-1-yl]anilino]-5-(oxan-4-ylamino)pyrazine-2-carboxamide, high SHBd values are due to the presence of multiple hydrogen bond donor groups, which enhance their inhibitory activity. For molecules like 3-[4-[4-(1-Methylpiperidin-4-yl)piperazin-1-yl]anilino]-5-(oxan-4-ylamino)-6-propan-2-ylpyrazine-2-carboxamide, high MLFER_S values reflect the presence of solvatophilic groups which improve solubility and interaction with the target protein. Basic groups (nBase), such as amines and piperidine rings, are prevalent in the identified molecules. For instance, the structure of 6-Ethyl-3-[4-[4-(4-methylpiperazin-1-yl)piperidin-1-yl]-3-propan-2-yloxyanilino]-5-(oxan-4-ylamino)pyrazine-2-carboxamide features several nitrogen atoms, contributing to its basicity. Molecules such as 6-(1-Methyl-3,6-dihydro-2H-pyridin-4-yl)-3-[4-[4-(4-methylpiperazin-1-yl)piperidin-1-yl]anilino]-5-(oxan-4-ylamino)pyrazine-2-carboxamide exhibit high MaxsssN values due to the presence of tertiary nitrogen atoms within piperazine rings. Finally, MLFER_BH sums the hydrogen bond basicity of all potential hydrogen bond acceptor sites. Compounds like pyrazinecarboxamide derivatives possess multiple hydrogen bond acceptor sites, enhancing their overall hydrogen bond basicity and binding affinity to the FLT3 tyrosine kinase.

### 2.8. Script-like Tool Description

To enhance the user experience with our model, an open-access script-based tool was created to automate the prediction of pIC_50_ and ${\mathrm{IC}}_{50}$ values for any compound using its SMILES code (Figure 4B). The tool can be accessed via the following link: https://github.com/Jacksonalcazar/Prediction-of-FLT3-Inhibitory-Activity (created on 6 July 2024). Designed to be user-friendly and efficient, this tool enables users to explore a list of SMILES codes in a simple, automated, and rapid manner, delivering results within seconds.

## 3. Materials and Methods

### 3.1. Data Curation

Data on FLT3 inhibitor compounds with published ${\mathrm{IC}}_{50}$ values were systematically extracted from the PubChem database [39,40] using the Requests library [41] and subsequently organized into a tabular format with the Pandas library [42] in Python 3. The dataset underwent rigorous cleaning, where duplicate entries were removed. We focused on compounds with ${\mathrm{IC}}_{50}$ values under 10 µM to prioritize higher potency in our analysis.

In the final stage of data preparation, we implemented feature scaling to standardize the range of independent variables, ensuring optimal performance of our ANN algorithms. This step was crucial for maintaining data integrity and ensuring compatibility with ANN-K and ANN-P, which are sensitive to the scale of the input data. We employed the standard scaler method from the scikit-learn library (sklearn) [43], which standardizes features by removing the mean and scaling to the unit variance. This normalization technique was applied to the training data using the fit_transform method, which computes the mean and standard deviation of each feature. Importantly, these parameters were then used to scale the test data using the transform method. This approach ensured that the model was not biased by any information from the test dataset, adhering strictly to the principles of statistical learning.

### 3.2. Molecular Descriptor Calculation

Initially, 1511 numerical molecular descriptors were computed using PaDEL-Descriptor 2.21 [44] and RDKit [28]. The dataset was curated to exclude descriptors which were either incompatible with all compounds or constant across the dataset, reducing the total number of descriptors to 1269. This curation was essential to ensure that all compounds had an equal number of descriptors, retaining only those most compatible with the molecular diversity and suitable for reliable modeling in subsequent machine learning analyses.

### 3.3. Benchmarking Machine Learning Methods with External Validation

The dataset, comprising 1350 compounds and 1269 descriptors, was imported using Python3 in conjunction with the Pandas library [42]. The experimental pIC_50_ values served as the target variables. To ensure a balanced representation of the dataset, we split it into training and testing sets at an 80:20 ratio using the train_test_split function from sklearn [43], with the random_state parameter set to 11 for reproducibility.

The machine learning models deployed in this study, implemented using sklearn [43], included the random forest regressor (RFR) [45], gradient boosting regressor (GBR) [46], support vector machine (SVM) [47], kernel ridge regression (KRR) [47], Gaussian process regressor (GPR) [48], and bagging with random forest (BRF) [49]. Additionally, an artificial neural network (ANN) architecture was implemented using Keras 2.13.1 (ANN-K) [50] and mirrored in PyTorch 2.4.0 (ANN-P) [51]. Consistency in random state settings was maintained across the applicable models. While the machine learning models were used with their default configurations, the ANN required a structured hyperparameter optimization process to achieve optimal performance.

#### 3.3.1. ANN Architecture

The ANN was designed as a sequential model consisting of three dense layers: a first layer with 500 neurons to handle the large number of features, an intermediate layer with 5 neurons for abstract data representations, and a final single-neuron output layer for pIC_50_ value regression. The ReLU activation function was used in the first two layers, with linear activation in the output layer and weights initialized using the HeNormal initializer. Data normalization was performed using StandardScaler from sklearn, and the model was trained with a batch size of 10 for 100 epochs to balance learning and prevent overfitting.

The choice of hyperparameters for the ANN model was based on computational efficiency and scalability to ensure a fair comparison with other machine learning methods. Specifically, the range of hyperparameters explored included layer1_sizes = [100, 300, 500], layer2_sizes = [1, 3, 5, 10, 15], epochs_list = [20, 50, 100, 120, 150], and batch_sizes = [5, 10, 20, 50]. This selection aimed to balance model complexity and computational cost, allowing the ANN to remain efficient and scalable while handling high-dimensional QSAR datasets. The results of this optimization process are provided in Table S1 of the Supporting Information, where the selected architecture demonstrated superior performance based on internal validation using a 90:10 split of the training data, with the R^2^ score as the evaluation metric.

#### 3.3.2. Model Evaluation and External Validation

Model performance was evaluated using the coefficient of determination (R^2^), mean absolute error (MAE), standard deviation (SD), and root mean squared error (RMSE) for both the training and testing datasets. These metrics were derived using the sklearn.metrics module, which offers robust tools for model evaluation. The testing datasets were specifically used for external validation, providing a comprehensive view of the predictive accuracy and error characteristics for each model, ensuring reproducibility was a fundamental aspect of this methodology. This was achieved by consistently using random seeds (set to 11) across the numpy, TensorFlow-Keras and PyTorch, and sklearn models, thereby maintaining a reliable and consistent assessment of performance.

### 3.4. Component Optimization Through Feature Selection

#### 3.4.1. Individual Descriptor Evaluation

To ascertain the influence of each molecular descriptor on the prediction of FLT3 inhibitor activity, analysis was conducted within the established framework (80:20 training:test split, random state = 11). Each descriptor was examined utilizing the RFR model, which was identified as the most effective in our earlier benchmarking. The evaluation centered on the coefficient of determination within the test set (R^2^ test). This metric was crucial as it quantitatively reflected the relevance of the descriptor, directly linking its presence to the precision of the model’s predictions.

#### 3.4.2. Analysis and Feature Selection Process

The next step entailed analyzing the top 100 descriptors using the R^2^ test metric to understand their correlation with FLT3 inhibitory activity. This analysis led to a selective inclusion of descriptors, starting with the most correlated one and progressively adding less correlated ones. This process aimed to find an optimal balance between model complexity and predictive accuracy.

### 3.5. Internal Validation

After the benchmarking phase and component optimization, the optimal model underwent internal validation using both the leave-one-out and 10 fold cross-validation techniques. The leave-one-out method, implemented via the LeaveOneOut class from the sklearn.model_selection module in Python, involves training the model on all data points except for one, which is reserved for testing. This process is systematically repeated for each data point in the dataset. In contrast, 10 fold cross-validation, implemented using the KFold class from the same module, divides the dataset into 10 subsets (folds), training the model on 9 folds and testing it on the remaining fold, iteratively covering all folds.

During this comprehensive validation process, the prediction accuracy of the model was quantified using the $R^{2}$ metric ($Q_{\mathrm{LOO}}^{2}$ for the leave-one-out method and $Q_{10-\mathrm{fold}}^{2}$ for 10 fold cross-validation). These key parameters enabled the comparison of the performance of our selected method with those of previous studies, ensuring its robustness beyond a fixed dataset.

### 3.6. Ligand-Based Virtual Screening

In the search for new and potential FLT3 tyrosine kinase inhibitors, a virtual screening was conducted using the PubChem database [39,40]. The analysis was performed by referencing structural similarity with the top 100 most active compounds, comparing each one individually. In other words, each of the top 100 most active compounds was compared using the Tanimoto coefficient [52] with 10.2 million molecules from PubChem [40,53], setting a similarity threshold of 90%. This analysis was carried out using the requests library and the similarity operation from PubChem’s PUG-REST data. Finally, the obtained SMILES codes were filtered, eliminating known FLT3 tyrosine kinase inhibitors.

The filtered list of SMILES codes was then processed through the model using the developed script. By predicting the pIC_50_ values, the model facilitated the prioritization of the five most promising compounds, streamlining the path toward experimental validation and accelerating the discovery of potent FLT3 tyrosine kinase inhibitors.

## 4. Conclusions

This study successfully demonstrated the applicability and efficacy of a QSAR-ML hybrid model in predicting the pIC_50_ values of FLT3 tyrosine kinase inhibitors based on the structural intricacies of ligands. This success was possible thanks to training with a wide variety of molecules, capturing the intrinsic factors involved in their activity. The comprehensive dataset, extensive molecular descriptor analysis, and meticulous benchmarking of various machine learning algorithms culminated in a model which showcased superior predictive capabilities based on its accuracy and simplicity.

Notably, the random forest regressor emerged as the most effective model, being validated through rigorous external and internal validation methods. This model serves as a simple and reliable tool for identifying potential FLT3 inhibitors, as evidenced by its $Q_{\mathrm{LOO}}^{2}$ value of 0.926 and a $Q_{10-\mathrm{fold}}^{2}$ value of 0.922 across a broad and heterogeneous dataset. Additionally, it demonstrated an R^2^ value of 0.941 and an SD of 0.237 in predicting the pIC_50_ values for 270 FLT3 tyrosine kinase inhibitor compounds outside of the training set.

Moreover, the component optimization and feature selection process highlighted the critical importance of specific molecular descriptors in FLT3 inhibitor efficacy, providing valuable insights into the structural features which influence inhibitor activity. This understanding facilitates the rational design of new FLT3 inhibitors, consequently streamlining the drug discovery process by focusing on compounds which exhibit these key structural characteristics.

Furthermore, the development of a user-friendly script-like tool for the prediction of pIC_50_ values represents a significant contribution to the cheminformatics toolbox, offering researchers a practical and efficient means of evaluating the FLT3 inhibitory potential of new compounds, including the application of ligand-based virtual screening. The tool’s scalability was demonstrated by its successful application to datasets containing up to 10.2 million molecules, underscoring its suitability for high-throughput screening scenarios. Additionally, it autonomously handles descriptor calculations and predictions, with built-in compatibility for RDKit and Open Babel formats, ensuring seamless workflows without requiring external platforms.

In summary, our study provides a simple model for predicting the pIC_50_ values of FLT3 tyrosine kinase inhibitors and sets a new benchmark in the integration of machine learning and QSAR methodologies for drug discovery. This approach offers enhanced predictive accuracy and user-friendly access, facilitating the rapid identification of new therapeutic candidates against AML via FLT3 inhibition. The scalability, efficiency, and compatibility of the developed tool further position it as a valuable resource for cheminformatics and early-stage drug discovery.

## Figures and Tables

**Table 1:** Performance comparison of machine learning models for predicting pIC_50_ values of FLT3 tyrosine kinase inhibitor compounds.

Metric and ML	RFR	GBR	KRR	GPR	BRF	ANN-K	ANN-P
R^2^ training	0.988	0.973	0.546	0.641	0.967	0.988	0.983
MAE training	0.082	0.126	0.489	0.469	0.136	0.070	0.082
SD training	0.102	0.154	0.638	0.526	0.172	0.101	0.121
RMSE training	0.102	0.154	0.638	0.568	0.172	0.103	0.123
R^2^ test	0.936	0.939	0.592	−0.228	0.931	0.907	0.895
MAE test	0.197	0.195	0.484	0.876	0.207	0.235	0.248
SD test	0.246	0.237	0.619	0.932	0.255	0.296	0.313
RMSE test	0.246	0.239	0.620	1.076	0.256	0.297	0.315

**Table 2:** Performance of random forest models for predicting pIC_50_ values of FLT3 tyrosine kinase inhibitor compounds based on 41 components.

	Training Set	Test Set
Size	1080	270
R^2^	0.989	0.941
MAE	0.081	0.193
SD	0.101	0.237
RMSE	0.101	0.238
$Q_{\mathrm{LOO}}^{2}$	0.926	
$Q_{10-\mathrm{fold}}^{2}$	0.922	

**Table 3:** Comparative performance of QSAR models for FLT3 inhibitors.

	Kar *^a^*	Shiha *^a^*	Abutayeha *^a^*	Bhujbala *^a^*	Fernandesa *^a^*	Ghosha *^a^*	This
	**(2012)**	**(2012)**	**(2019)**	**(2020)**	**(2020)**	**(2021)**	**Work**
Dataset size	67	72	93	63	40	40	1350
Train set size	51	25	76	45	28	30	1080
Test set size	16	47	17	18	12	10	270
R^2^ training	0.956	0.98	0.86	0.956	0.80	0.983	0.989
R^2^ test	0.891	0.76	0.57	0.707	0.80	0.698	0.941
SD test	0.435	0.66	-	>0.895	0.31	0.452	0.237
$Q_{\mathrm{LOO}}^{2}$	0.747	0.58	0.65	0.57	0.60	0.802	0.926

*^a^* Data obtained from  [10,11,12,13,14,15].

**Table 4:** Names and characterizations of the five most important descriptors for the model’s development, ordered by priority.

Priority	Descriptor	Name	Description
1°	SHBdb [32,33]	Sum of E-states for (strong) hydrogen bond donors	The value is calculated as the sum of each atom capable of donating a hydrogen atom, weighted by its electronic environment and topological position (E-state).
2°	MLFER_S [33,34]	Molecular linear free energy relation_S	Cumulative sum of the free energy contributions of the solvatophilic groups in a molecule, calculated using previously established empirical values for their interactions with solvents.
3°	nBase	Number of basic groups	Number of basic groups in the molecule, especially nitrogenous groups.
4°	MaxsssN [32,35]	Maximum atom-type E-state: > N-	Maximum electrotopological state present in nitrogen atoms with three single bonds.
5°	MLFER_BH [34,36]	Overall or summation solute hydrogen bond basicity	Total hydrogen bond basicity in a molecule calculated by summing the contributions of all possible hydrogen bond acceptor sites in the molecule.

**Table 5:** Top five candidates for FLT3 inhibitors identified by ligand-based virtual screening.

IUPAC Name	Structure	pIC_50_
6-Ethyl-3-[3-methoxy-4-[4-(1-methylpiperidin-4-yl)piperazin-1-yl]anilino]-5-(oxan-4-ylamino)pyrazine-2-carboxamide	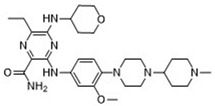	9.34
6-Ethyl-3-[3-methoxy-4-[4-(4-propan-2-ylpiperazin-1-yl) piperidin-1-yl]anilino]-5-(oxan-4-ylamino)pyrazine-2-carboxamide	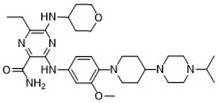	9.34
3-[4-[4-(1-Methylpiperidin-4-yl)piperazin-1-yl]anilino]-5-(oxan-4-ylamino)-6-propan-2-ylpyrazine-2-carboxamide	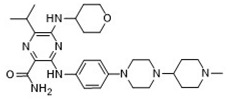	9.29
6-(1-Methyl-3,6-dihydro-2H-pyridin-4-yl)-3-[4-[4-(4-methylpiperazin-1-yl)piperidin-1-yl]anilino]-5-(oxan-4-ylamino)pyrazine-2-carboxamide	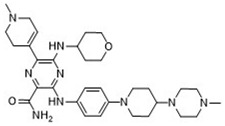	9.27
6-Ethyl-3-[4-[4-(4-methylpiperazin-1-yl)piperidin-1-yl]-3-propan-2-yloxyanilino]-5-(oxan-4-ylamino)pyrazine-2-carboxamide	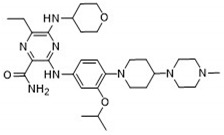	9.27

## Data Availability

We have made the prediction model publicly available. One can access the script at the following GitHub repository: https://github.com/Jacksonalcazar/Prediction-of-FLT3-Inhibitory-Activity (accessed on 10 December 2024).

## Supplementary Materials

The following supporting information can be downloaded at https://www.mdpi.com/article/10.3390/ph18010096/s1. Table S1: Summary of ANN-P configurations and their corresponding score values from internal validation.
